# KDELR2 as a diagnostic and prognostic biomarker of bladder urothelial carcinoma and its correlation with immune infiltration

**DOI:** 10.1590/1678-4685-GMB-2023-0002

**Published:** 2023-09-25

**Authors:** Sai Ma, Longqi Sa, Jitao Zhang, Kuo Jiang, Baoguo Mi, Lequn Shan

**Affiliations:** 1 Air Force Medical University, School of Stomatology, National Clinical Research Centre for Oral Disease, State Key Laboratory of Military Stomatology, Department of Prosthodontics, Shaanxi Key Laboratory of Stomatology, Xi’an, Shaanxi, China.; 2 Xi’an Jiaotong University, Honghui Hospital, Department of Spine Surgery, Xi’an, Shaanxi, China.

**Keywords:** Bladder Urothelial Carcinoma (BLCA), Bioinformatic analysis, Immune infiltration, KDELR2

## Abstract

KDELR2 has been reported as a promotive factor for the genesis and progression of several malignancies. However, it is uncertain how it affects bladder urothelial carcinoma (BLCA). Using data extracted from online databases, an enhanced expression of KDELR2 in BLCA tissues was verified. Overexpression of KDELR2 was correlated with advanced clinicopathologic characteristics and unfavourable prognosis of BLCA. Receiver operating characteristic analysis highlighted the potential diagnostic value of KDELR2. Univariate and multivariate logistic regression analyses further revealed the predictive effect of KDELR2 for the prognosis of BLCA. KDELR2 was primarily enriched in biological functions related to organization of the extracellular matrix. TIMER, ssGSEA and GEPIA analyses suggested that KDELR2 expression is positively related to the infiltration of macrophages, Th2 cells and neutrophils. Finally, knocking-down of KDELR2 in T24 cells resulted in reduced proliferation, migration and macrophages recruitment. These results suggest that KDELR2 overexpression is an indicator for poor prognosis of BLCA and it has the potential to be employed as an immunotherapy target for BLCA.

## Introduction

BLCA is a type of cancer that influences a large number of populations around the world and requires expensive care (Diamandis et al., 2010). In 2021, there were 83,730 new patients and 17,200 related deaths in the US alone, and these numbers were slightly higher than those in 2020 (Laguna, 2019; Siegel et al., 2021).

Generally, BLCA is a heterogeneous malignancy, and patients may show different responsiveness to therapies due to differences in their underlying basic biology and various host-related factors. In general, BLCA can be categorized into two types (Kang et al., 2020). The muscle invasive type is very lethal and death may occur within two years of diagnosis if it is left untreated. For the less life-threatening nonmuscle invasive type, more than 45% of the patients will experience recurrence or progression of the disease at some point in their lives (Sylvester et al., 2006). Such a high recurrence and progression rate poses an enormous challenge for doctors and imposes a great financial burden on patients (Abdollah et al., 2013; Berdik, 2017; James and Gore, 2013). 

Despite great improvements in surgical techniques, immunotherapy, radiotherapy and perioperative chemotherapy, the long-term prognosis of BLCA remains dismal (Witjes et al., 2021). To facilitate decision-making regarding the diagnosis, prognosis and treatment of BLCA, we need to explore molecular mechanisms underlying the tumorigenesis and development of the disease.

The KDEL receptors, which contain three subtypes, are mainly involved in the retrieve of chaperones from the Golgi complex to the endoplasmic reticulum (Hsu et al., 1992; Capitani and Sallese, 2009; Kokubun et al., 2019). In addition to their chaperone-retrieval activity, recent studies suggest that KDEL receptors have several other functions, such as ER quality control mediated through the MAPK pathway and signal transduction abilities mediated through the Src pathway (Yamamoto et al., 2003; Pulvirenti et al., 2008; Cancino et al., 2013). 

Since MAPK and Src pathways are widely involved in various cellular functions, the regulating role of KDELRs in growth, survival, autophagy, cytoskeletal remodelling of cells, as well as its influence on immune responses, have been postulated and proven by many recent studies. Interestingly, a regulatory function of KDELR in tumorigenesis and progression has also been observed. For example, several published researches suggested that KDELR2 can be a promotive factor for the tumorigenesis of glioblastoma (Liao et al., 2019; Mao et al., 2020) and breast cancer (Wei et al., 2021). In non-small cell lung cancer, KDELR2 can enhance the secretion of matrix metalloproteases and thus promote tumor invasion and metastasis (Bajaj et al., 2020). However, it remains obscure whether KDELR2 is also involved in BLCA development.

To explore whether KDELR2 is also involved in BLCA, comprehensive bioinformatics analysis of several public databases was performed. Furthermore, *in vitro* studies using T24 cells were performed to validate the influences of KDELR2 on proliferation, migration and macrophages recruiting ability of the cells. This bioinformatic and *in vitro* study will elucidate the potential diagnostic, prognostic and therapeutic value of KDELR2 for BLCA.

## Material and Methods

### Acquisition of patient sample data

From the TCGA database (https://cancergenome.nih.gov), we retrieved genome-wide profiling data (including 414 tumour samples and 19 adjacent nontumor samples) and corresponding information about clinical and pathological characteristics of BLCA patients. Patients in the TCGA data base were divided into low- and high- KDELR2 expression groups using the median value of KDELR2 expression as the cutoff point. By using the GEO database (https://www.ncbi.nlm.nih.gov/geo/), we acquired transcriptional profiles of several BLCA cohorts, including GSE188715 (BLCA samples: 57; paired adjacent nontumor samples: 13), GSE3167 (BLCA samples: 51; paired adjacent nontumor samples: 9) and GSE32894 (308 BLCA samples). To confirm the different expression pattern of KDELR2 at the protein level, information from the Human Protein Atlas database (http://www.proteinatlas.org/) was analysed. Information regarding to gene amplification and mutation of KDELR2 was obtained from the cBioPortal for Cancer Genomics (http://www.cbioportal.org/).

### Receiver operating characteristic (ROC), univariate and multivariate logistic regression analyses

The pROC package (https://cran.r-project.org/web/packages/pROC/) was used to draw ROC curves. Univariate Cox regression was used to evaluate whether KDELR2 expression is correlated with the overall survival (OS) of BLCA patients. We also performed multivariate analysis to verify whether KDELR2 is an independent prognostic indicator for survival. Cox P value lower than 0.05 was used as significance threshold.

### Kaplan-Meier analysis

The correlation between KDELR2 expression and OS, disease-specific survival (DSS) and progression-free survival (PFS) was evaluated by drawing the Kaplan‒Meier survival curves. The hazard ratio (HR) and log-rank p value of the 95% confidence interval were calculated.

### Construction and evaluation of the nomogram for survival prediction

A nomogram for prediction of OS probability in BLCA patients was constructed using information about KDELR2 expression and clinicopathological features (Shen et al., 2020). The accuracy of the nomogram was evaluated by drawing a correction curve.

### Differentially expressed gene identification and functional enrichment analysis

Altogether, 414 BLCA patients were separated into two groups based on the different expression levels of KEDLR2. Using the LIMMA package in R version 3.6.3 (http://www.R-project.org/) (Ritchie et al., 2015), differentially expressed genes (DEGs) were identified. The adjusted p-values were obtained through multiple testing using the BH method, which better controls the false positive rate. DEGs are defined as genes that showed adjusted p values lower than 0.05 and |log2(Fold Change)| values equal to or higher than 1 (Yu et al., 2012). Gene Ontology (GO) enrichment analysis was conducted with respect to biological process, molecular function, and cellular component and Kyoto Encyclopedia of Genes and Genomes (KEGG) was used to perform pathway enrichment analysis. The findings were visualized through the ggplot2 package.

### Gene Set Enrichment Analysis (GSEA)

GSEA was performed to explore whether there were significant and concordant differences in a previously defined set of genes between the low- and high-KDELR2 expression groups. Significant enriched genes were defined as those showing an NOM p-value lower than 0.05 and FDR q-value lower than 0.25.

### Protein-protein interaction network construction

Through the Search Tool for the Retrieval of Interacting Genes/Proteins (STRING) database (https://string-db.org/), the direct and indirect protein‒protein interaction network of KDELR2 was analysed (Szklarczyk et al., 2019). Confidence scores higher than 0.4 were considered to have median significance.

### Analysis of immune infiltration

For single-sample GSEA (ssGSEA), gene markers for immune cells were acquired from the published literature (Bindea et al., 2013) and the visualization of the findings was realized by ggplot2 package. Systematical analysis of immune infiltrates was also performed using the TIMER database (http://timer.cistrome.org) (Li et al., 2020). Then, gene expression correlation analysis was performed in GEPIA (http://gepia.cancer pku.cn/index.html) to further verify the results (Li *et al.*, 2021). The correlation coefficients were calculated using the Spearman method.

### Cell culture and transfection

The widely used T24 BLCA cell line was used in the present study to further verify the role of KDELR2 in development of bladder cancer (Zhang et al., 2023). The cells were cultured in Dulbecco’s modified Eagle’s medium containing 10% fetal bovine serum. Small-interfering RNAs (siRNAs) against KDELR2 (Sangon Biotech, Shanghai, China) were used to modify the expression of KDELR2. Lipofectamine 2000 (Thermo Fisher Scientific) was used for transfection. Cell Counting Kit-8 (CCK-8) assay was performed to assess the proliferation capacity of the cells.

### Gene and protein expression assays

Forty-eight hours after transfection, mRNAs were extracted and reverse transcription reactions were performed. Thereafter, the level of KDELR2 mRNA was evaluated using quantitative real-time polymerase chain reaction (SYBR® Premix Ex Taq™, TaKaRa, Shiga, Japan). Beta-actin was included for normalization. For western blotting, the cells were first treated with RIPA buffer. Then the protein samples were separated and transferred onto polyvinylidene fluoride membranes. The membranes were then subjected for blocking and incubated with antibodies against KDELR2 (Abcam) or beta-actin (Abcam) at 4 °C overnight. After washing, secondary antibodies were used for incubation of the samples. The staining patterns were analysed with the FluorChem FC2 system (Alpha Innotech).

### Wound healing assay

The cells were cultured for 24 hours in a 6-well plate with 5 x 10^5^ cells per well. A straight line was scraped into the cell layer using a 1 mm pipette tip. Microscopic observation was performed at 0 h and 24 h after scraping.

### Transwell migration and macrophage recruitment assay

T24 cells (2 x 10^5^ per well) transfected with control siRNA or si-KDELR2 were seeded in the upper chambers of Transwell plates (Millipore) in FBS-free medium. In the lower chambers, DMEM with 10% FBS was used to attract the cells for migration. After 24 hours of incubation, fixing and staining of the cells that passed through the membrane was performed. Then, we removed the cells on the membrane’s upper surface and observed the migrated cells under a microscope. For macrophage recruitment assay, macrophages differentiated from THP-1 human monocyte by PMA induction were seeded in the top chambers of Transwell plates. T24 cells were placed in the chamber below the cell permeable membrane. After 24 hours of incubation, the migrated cells were fixed and stained for microscopic observation.

### Statistical analysis

For bioinformatics analysis, R version 3.6.3 was used. Student’s t-test was used to evaluate the differential expression of KDELR2 in cancer tissues and normal controls. It was also used to compare KDELR2 mRNA level and proliferation capacity of T24 cells between the control group and siKDELR2 group in *in vitro* studies. Wilcoxon rank-sum test and Kruskal‒Wallis test was used to investigate the possibility of a connection between the clinicopathological characteristics of BLCA patients and the expression of KDELR2. In the ROC curve analysis, the area under the curve (AUC) was calculated to indicate diagnostic accuracy. The log-rank test was used for Kaplan‒Meier survival analysis. Univariate and multivariate Cox analyses were used to screen for potential prognostic factors. The Spearman correlation test was used to study the correlation between KDELR2 expression and the infiltration of immune cells. In all analyses, ns, *, * *, and * * * indicate p > 0.05, p < 0.05, p < 0.01 and p < 0.001, respectively.

## Results

### KDELR2 is highly expressed in BLCA tissues

**Figure 1 -:**
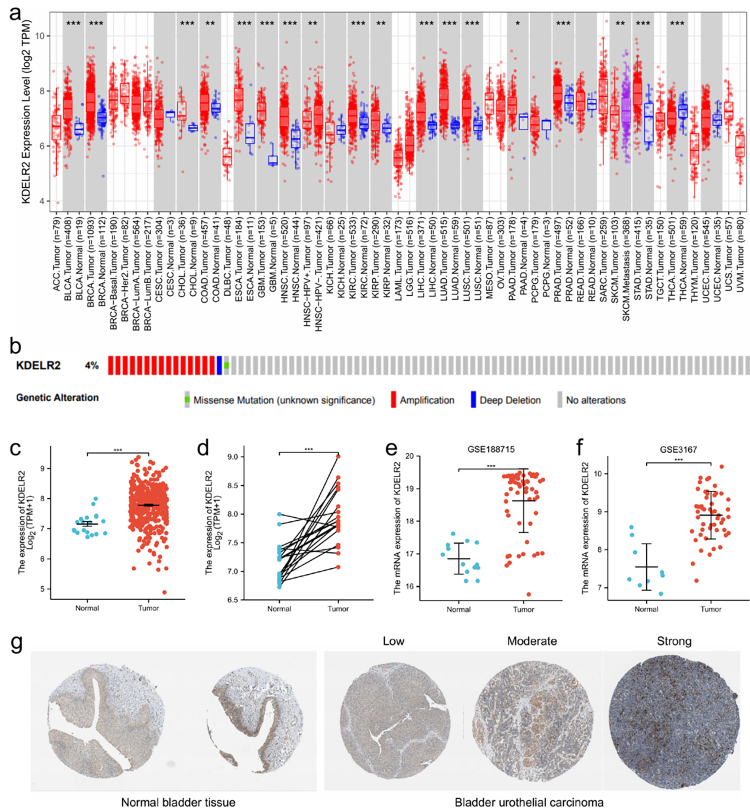
The expression of KDELR2 was upregulated in various malignancies, including BLCA, and the high expression of KDELR2 was validated using the GEO and HPA databases. (a) Expression level of KDELR2 in different types of human malignancies from the TIMER database. (b) The cBioPortal OncoPrint map showing the distribution of KDELR2 genome changes in BLCA patients. (c) Expression level of KDELR2 in normal tissues and tumour tissues from BLCA patients. (d) Expression level of KDELR2 in paired adjacent tissues and tumour tissues from BLCA patients. (e, f) Expression of KDELR2 in tumour and normal tissues from the GSE188715 and GSE3167 datasets in the GEO database. (g) Increased expression of KDELR2 in BLCA was also validated at the protein level using the HPA database (immunohistochemistry).

KDELR2 expression was enhanced in various tumour tissues, including breast cancer, head and neck cancer, kidney cancer, liver cancer, lung cancer, colon cancer, bladder cancer and glioblastoma (Figure 1a). On the other hand, KDELR2 expression was reduced in thyroid carcinoma (Figure 1a). Furthermore, the genome and copy number of KDELR2 were analysed to explore the mutation level of KDELR2 in BLCA. The cBioPortal map data revealed that approximately 4% of BLCAs had gene amplification or missense mutations in KDELR2.

The different expression levels of KDELR2 were also verified at mRNA level. KDELR2 expression was increased in BLCA tissues in comparison with normal control (p< 0.001) (Figure 1c). Such an enhanced expression of KDELR2 was further confirmed in BLCA tissues and their matched paracarcinoma tissues (p< 0.001, Figure 1d).

Analysis of two additional independent external GEO datasets (validation cohorts, GSE188715 and GSE3167) further validated the higher level of KDELR2 in BLCA tissues (p<0.001, Figure 1e-f). Using information from Human Protein Atlas database, we further verified the results at protein level (Figure 1g).

### Expression of KDELR2 is associated with clinicopathological characteristics of BLCA

**Figure 2 -:**
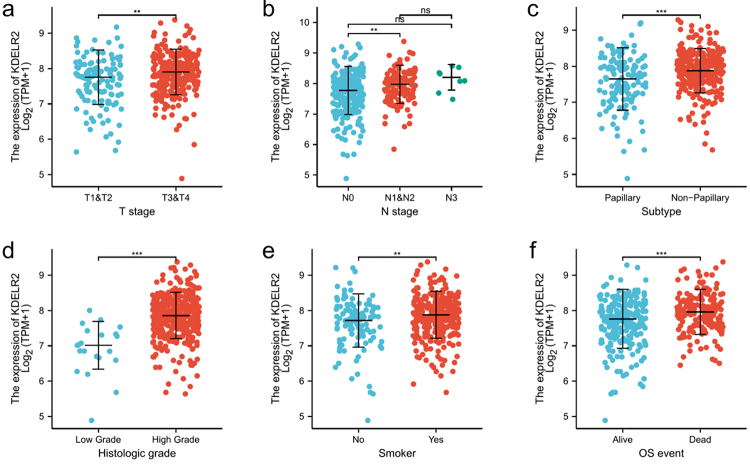
Expression of KDELR2 is correlated with poor clinicopathological features in BLCA patients. A higher expression level of KDELR2 is associated with poor clinical clinicopathological features in BLCA in TCGA datasets [T stage (a), N stage (b), histological subtype (c), histological grade (d), smoking habits (e) and OS events (f)]. **p < 0.01, ***p < 0.001.

Table 1 shows the clinicopathological features of 408 BLCA cases obtained from the TCGA database. These cases were categorized into high- and low-KDELR2 expression groups. Significant association was found between increased KDELR2 expression and poor characteristic for clinical stage, N stage, tumour histological type, histological grade, smoking status, and OS events (Figures 2a-f and Table 1).

**Table 1 -:** Clinical characteristics of patients with high or low expression levels of KDELR2 from the TCGA dataset.

Characteristics	Low expression of KDELR2	High expression of KDELR2	p
n	204	204	
T stage, n (%)			0.044
T1	1 (0.3%)	2 (0.5%)	
T2	70 (18.7%)	49 (13.1%)	
T3	90 (24.1%)	104 (27.8%)	
T4	23 (6.1%)	35 (9.4%)	
N stage, n (%)			0.011
N0	131 (35.8%)	106 (29%)	
N1	16 (4.4%)	30 (8.2%)	
N2	31 (8.5%)	44 (12%)	
N3	2 (0.5%)	6 (1.6%)	
M stage, n (%)			0.534
M0	14 (55.1%)	82 (39.6%)	
M1	5 (2.4%)	6 (2.9%)	
Pathologic stage, n (%)			0.295
Stage I	1 (0.2%)	1 (0.2%)	
Stage II	85 (20.9%)	45 (11.1%)	
Stage III	66 (16.3%)	74 (18.2%)	
Stage IV	51 (12.6%)	83 (20.4%)	
Primary therapy outcome, n (%)			0.295
PD	31 (8.8%)	37 (10.5%)	
SD	13 (3.7%)	16 (4.6%)	
PR	9 (2.6%)	13 (3.7%)	
CR	128 (36.5%)	104 (29.6%)	
Gender, n (%)			0.115
Female	46 (11.3%)	61 (15%)	
Male	158 (38.7%)	143 (35%)	
Age, n (%)			0.194
<=70	122 (29.9%)	108 (26.5%)	
>70	82 (20.1%)	96 (23.5%)	
Histologic grade, n (%)			<0.001
High Grade	183 (45.2%)	201 (49.6%)	
Low grade	20 (4.9%)	1 (0.2%)	
Subtype, n (%)			0.002
Non-papillary	121 (30%)	150 (37.2%)	
Papillary	81 (20.1%)	51 (12.7%)	
Lymphovascular invasion, n (%)			0.040
No	72 (25.6%)	58 (20.6%)	
Yes	64 (22.8%)	87 (31%)	
Smoke, n (%)			0.008
No	67 (17%)	42 (10.6%)	
Yes	131 (33.2%)	155 (39.2%)	
Age, median (IQR)	67 (59.75, 75.25)	70 (61, 77)	0.126

PD: Progressive disease; SD: Stable disease; PR: Partial response; CR: Complete response;

IQR: Interquartile range

### Expression of KDELR2 is associated with prognosis of BLCA patients

As indicated by univariate Cox analysis, for patients with BLCA, advanced pathologic grade and stage, lymphovascular invasion and high KDELR2 expression were negatively correlated with OS (Table 2). Multivariate regression analysis indicated that KDELR2 was an independent prognostic factor for OS in BLCA patients (HR = 2.901, 95% CI = 1.475-5.705, p = 0.002, Table 2).

**Table 2 -:** Univariate and multivariate Cox regression analyses of the correlation between clinical characteristics and OS in BLCA patients in the TCGA dataset.

Characteristics	Total (n)	Univariate analysis		Multivariate analysis	
		HR (95% CI)	*P*	HR (95% CI)	*P*
Gender (male vs. female)	413	0.849 (0.616-1.169)	0.316		
Age (>70 vs. <=70)	413	1.421 (1.063-1.901)	0.018	1.117 (0.594-2.102)	0.730
T stage (T3/T4 vs. T1/T23)	379	2.199 (1.515-3.193)	<0.001	1.335 (0.281-6.347)	0.716
N stage (N1/N2/N3 vs. N0)	369	2.289 (1.678-3.122)	<0.001	0.875 (0.371-2.064)	0.760
M stage (M1 vs. M0)	213	3.136 (1.503-6.544)	0.002	0.732 (0.209-2.565)	0.626
Pathologic stage (stage III/IV vs. Stage I/II)	411	2.310 (1.596-3.342)	<0.001	1.484 (0.231-9.540)	0.678
Primary therapy outcome (PD/SD vs. PR/CR)	357	0.226 (0.162-0.315)	<0.001	0.306 (0.144-0.652)	0.002
Smoker (yes vs. no)	400	1.305 (0.922-1.847)	0.133		
Histologic grade (high grade vs. low grade)	410	2.972 (0.735-12.008)	0.126		
Lymphovascular invasion (yes vs. no)	282	2.294 (1.580-3.328)	<0.001	1.952 (0.853-4.463)	0.113
KDELR2 (high vs. low413)	413	1.567 (1.163-2.112)	0.003	2.901 (1.475-5.705)	0.002

HR: Hazard ratio

**Figure 3 -:**
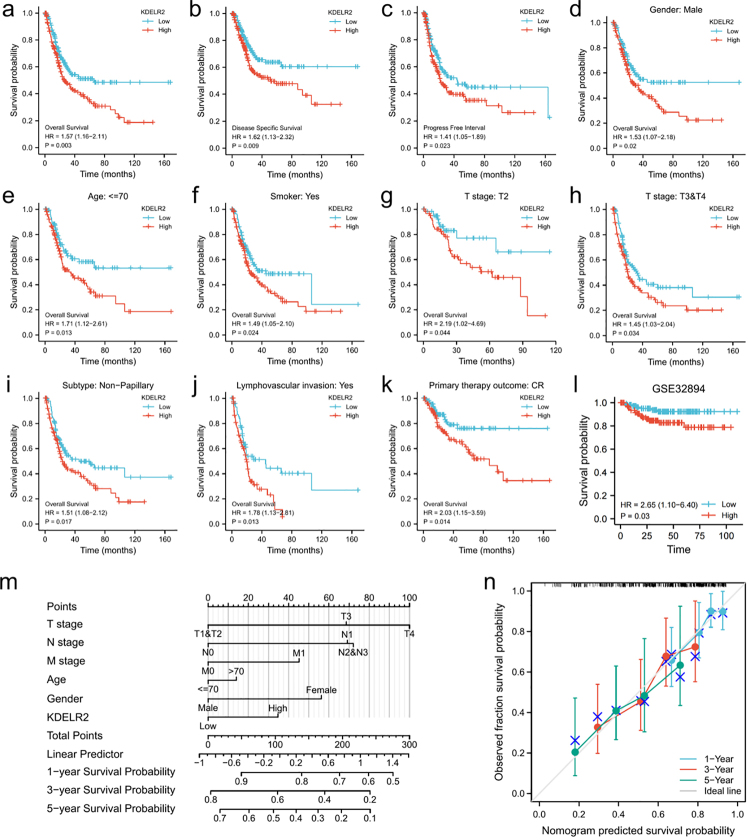
Kaplan‒Meier survival curve analysis of the prognostic significance of KDELR2 in BLCA. (a-c) Kaplan‒Meier survival curve analysis of OS, DSS and PFI in the TCGA dataset. Subgroup analysis for male patients (d), age less than 70 years (e), smokers (f), T2 (g), T3&T4 (h), nonpapillary subtype (i), accompanied by lymphovascular invasion (j), and complete response (CR) (k). (l) The association between KDELR2 expression and the OS of BLCA patients was also validated in the GEO dataset GSE32894. (m) Nomogram chart for predicting 1-, 3-, and 5-year overall survival. (n) Calibration plot of the nomogram for OS prediction.

PD: Progressive disease; SD: Stable disease; PR: Partial response; CR: Complete response;

It was revealed by Kaplan‒Meier survival analyses that BLCA patients showing higher KDELR2 expression had shorter OS, DSS, and PFI (Figure 3a-c). In addition, the correlation between KDELR2 overexpression and shorter OS was verified by subgroup analysis (Figure 3d-k). We also analysed the GEO dataset (GSE32894) to validate the reproducibility of the KDELR2 expression data in BLCA patient prognosis.

With the purpose to predict the survival probability of BLCA patients, we constructed a nomogram using KDELR2 expression data and clinical variables. It was indicated that KDELR2 expression level was a better predictive factor than the traditional clinical features like age (Figure 3m). In addition, the predicted and observed values were well aligned on the calibration plot (Figure 3n).

### Diagnostic value of KDELR2 in BLCA patients

**Figure 4 -:**
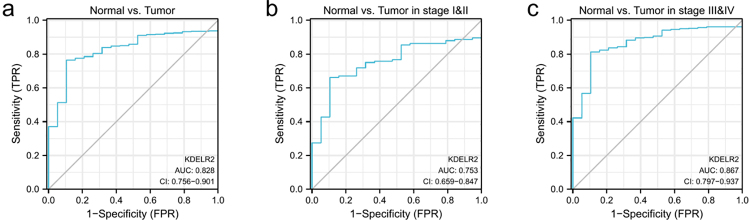
Diagnostic value of KDELR2 expression in BLCA. (a) ROC curve analysis for KDELR2 expression in BLCA and adjacent tissue. (b, c) ROC curve analysis for KDELR2 expression in BLCA patients in stages I&II or III&IV.

ROC analysis was conducted to assess whether KDELR2 expression level can be used to differentiate BLCA tissues from nontumor tissues. The estimated AUC was 0.828 (95% CI: 0.756-0.901, Figure 4a), indicating a relatively high diagnostic value of KDELR2. Furthermore, ROC analysis was also performed in subgroups of BLCA patients in different stages. The AUC was 0.753 for stages I and II (95% CI: 0.659-0.847, Figure 4b) and 0.867 for stages III and IV (95% CI: 0.797-0.937, Figure 4c). 

### Predicted biological function and pathways of KDELR2 in BLCA

**Figure 5 -:**
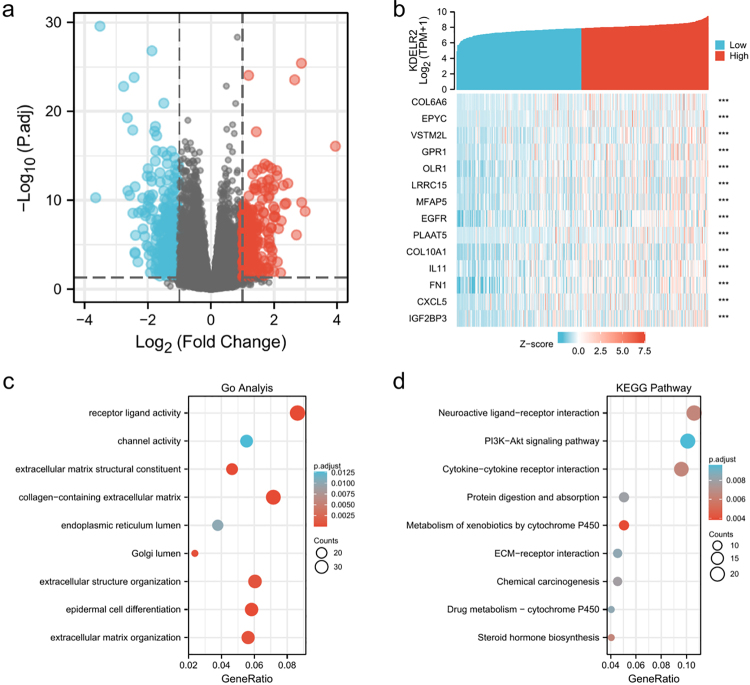
GO and KEGG enrichment analyses of DEGs related to KDELR2 in BLCA. (a) Volcano plot of DEGs associated with KDELR2 expression. (b) Heatmap of DEGs associated with the expression of KDELR2. (c) GO enrichment analysis showing the BP (biological processes), CC (cellular components), and MF (molecular function) of genes co-expressed with KDELR2. (d) Significantly enriched KEGG terms obtained from KEGG enrichment analysis of genes co-expressed with KDELR2. *** p < 0.001

**Figure 6 -:**
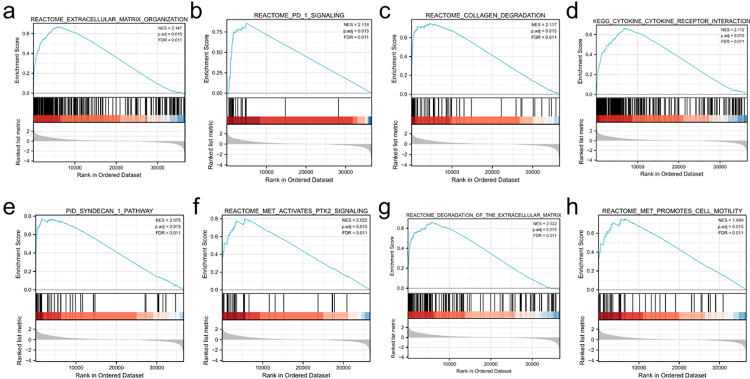
Enrichment plots from GSEA. DEGs related to KDELR2 were significantly enriched in extracellular matrix organization (a), PD-1 signalling (b), collagen degradation (c), cytokine receptor interaction (d), syndecan 1 pathway (e), MET-activated PTK2 signalling (f), degradation of the extracellular matrix (g), and MET-promoted cell motility (h) pathways. NES, normalized enrichment scores; FDR, false discovery rate.

Altogether, 541 DEGs were found. Among them, 262 genes were upregulated and 279 were downregulated (Figure 5a, b). For biological process (BP) in GO term analysis, it was found that epidermal development, skin development and epidermal cell differentiation were enriched. For cellular component (CC), it was revealed that collagen-containing extracellular matrix was enriched. GO term analysis for molecular function (MF) showed that receptor ligand activity and extracellular matrix structural constituents were significantly enriched. It was revealed by KEGG analysis that neuroactive ligand‒receptor interactions, PI3K-Akt signalling pathways and cytokine‒cytokine receptor interactions were enriched (Figure 5c-d). In addition, KDELR2 related signalling pathways were predicted by GSEA analysis using the MsigDB collection. The enriched pathways included extracellular matrix organization (Figure 6a), PD-1 signalling (Figure 6b), collagen degradation (Figure 6c), cytokine receptor interaction (Figure 6d), syndecan1 pathway (Figure 6e), MET-activated PTK2 signalling (Figure 6f), degradation of the extracellular matrix (Figure 6g) and MET-promoted cell motility (Figure 6h).

### KDELR2-related protein network in BLCA tissue

Ten proteins that exhibited intertwined interactions with KDELR2 were identified. The gene names for the identified proteins and their annotation scores were listed in the supplementary figure. The top ten KDELR2-interacting genes included COPA, ARFGAP1, ARFGAP3, COPB1, ARF1, KDELR3, ASAP1, CLTA, KDELR1, and ASAP2.

### KDELR2 expression was related with immune infiltration in BLCA

**Figure 7 -:**
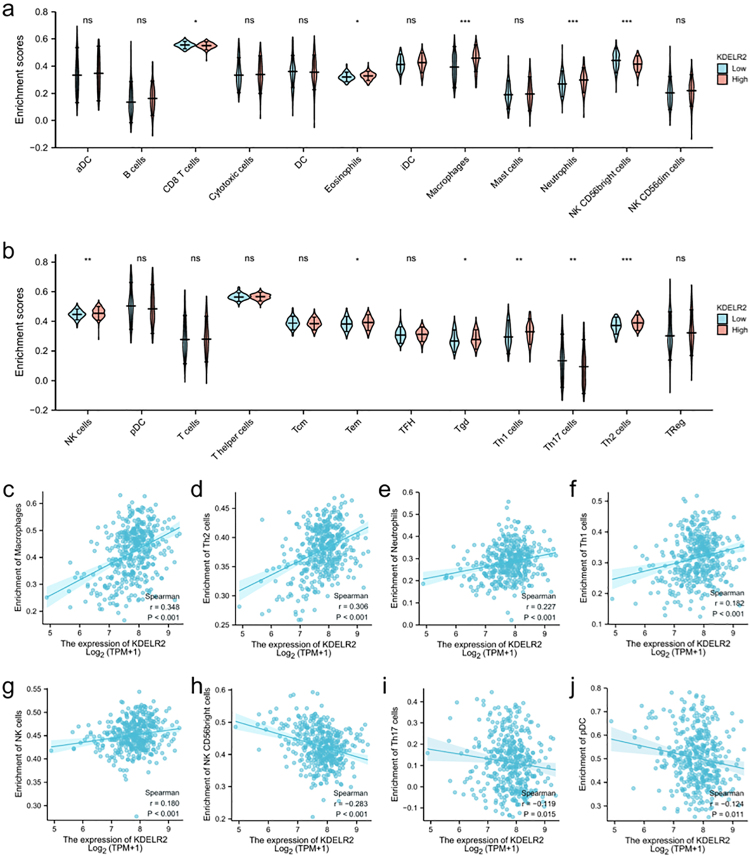
Correlation analysis of KDELR2 expression and immune infiltration in BLCA. (a, b) Differential distribution of immune cells in patients with high or low KDELR2 expression. The expression level of KDELR2 was positively related to the infiltration levels of macrophages (c), Th2 cells (d), neutrophils (e), Th1 cells (f), and NK cells (g) and negatively related to the infiltration of NK CD56^bright^ cells (h), Th17 cells (i), and pDCs (j). *p < 0.05, **p < 0.01, ***p < 0.001, ns, no significance.

It was revealed by ssGSEA that enhanceed KDELR2 expression was correlated with higher infiltration of eosinophils, macrophages, neutrophils and Th2 cells in BLCA patients. In contrast, the infiltration levels of CD8+ T cells, NK CD56^bright^ cells and Th17 cells were lower in the KDELR2 overexpression group (Figures 7a, b). It was also found that KDELR2 expression was positively corelated with the infiltration of macrophages (Figure 7c), Th2 cells (Figure 7d), neutrophils (Figure 7e), Th1 cells (Figure 7ff) and NK cells (Figure 7g). On the other hand, KDELR2 expression was negatively corelated with the abundance of infiltrating NK CD56^bright^ cells (Figure 7h), pDCs (Figure 7i) and Th17 cells (Figure 7j). In addition, tumor-infiltrating immune cell was also studied using the TIMER and GEPIA databases based on sets of immunological markers. The results were adjusted based on tumour purity, and a significant correlation between KDELR2 expression and markers for Treg, monocyte, and TAM sets were identified (Table 3).

**Table 3 -:** Correlation analysis between KDELR2 expression and markers of immune cells based on TIMER and GEPIA analysis.

Cell Type	Gene Marker	None Cor	P	Purity Cor	p	Tumor R	p	Normal R	P
B cell	CD19	0.132	**	0.083	0.111	0.16	**	0.038	0.69
	CD20 (KRT20)	0.128	*	0.177	**	0.083	0.096	-0.055	0.56
	CD38	0.142	**	0.095	0.068	0.21	***	0.23	*
CD8^+^ T cell	CD8A	0.102	*	0.038	0.469	0.14	**	-0.019	0.84
	CD8B	0.07	0.157	0.026	0.616	0.1	*	-0.14	0.14
Tfh	BCL6	0.003	0.952	-0.003	0.947	0.071	0.15	-0.15	0.13
	ICOS	0.126	*	0.067	0.199	0.16	***	0.19	*
	CXCR5	0.134	**	0.073	0.164	0.092	0.065	0.089	0.35
Th1	T-bet (TBX21)	0.098	*	0.035	0.498	0.13	**	0.29	0.23
	STAT4	0.046	0.344	-0.027	0.604	0.13	**	0.27	0.27
Th1	IL12RB2	0.135	**	0.106	*	0.2	***	0.26	0.28
	WSX1 (IL27RA)	0.225	***	0.198	***	0.27	***	0.21	0.38
	STAT1	0.214	***	0.178	***	0.29	***	0.4	0.091
	IFN-γ (IFNG)	0.069	0.165	0.018	0.733	0.11	*	0.19	0.43
	TNF-a (TNF)	0.126	*	0.107	*	0.2	***	-0.25	0.29
Th2	GATA3	0.049	0.624	0.096	0.067	0.032	0.52	0.37	0.12
	CCR3	0.196	***	0.202	***	0.25	***	0.37	0.12
	STAT6	-0.073	0.143	-0.046	0.378	0.025	0.62	0.15	0.54
	STAT5A	0.182	***	0.159	**	0.24	***	-0.14	0.57
Th9	TGFBR2	0.28	***	0.159	**	0.24	***	-0.14	0.11
	IRF4	0.119	*	0.042	0.425	0.14	**	0.19	0.43
	PU.1 (SPL1)	0.166	***	0.108	*	0.2	***	0.086	0.73
Th17	STAT3	0.229	***	0.195	***	0.32	***	0.29	0.23
	IL-21R	0.164	***	0.112	*	0.2	***	0.2	0.41
	IL-23R	-0.019	0.698	-0.032	0.545	0.022	0.66	0.21	0.38
	IL-17A	-0.092	0.064	-0.093	0.075	-0.061	0.22	-0.22	0.38
Th22	CCR10	0.106	*	0.089	0.086	0.14	**	0.061	0.81
	AHR	0.175	***	0.215	***	0.2	***	0.27	**
Treg	FOXP3	0.164	***	0.128	*	0.2	***	0.17	0.5
	CD25 (IL2RA)	0.214	***	0.178	***	0.26	***	-0.023	0.93
	CCR8	0.281	***	0.266	***	0.3	***	0.44	0.057
T cell exhaustion	PD1 (PDCD-1)	0.086	0.082	0.017	0.749	0.13	**	0.14	0.57
	CTLA4	0.088	0.078	0.02	0.698	0.14	**	0.088	0.72
	LAG3	0.096	0.053	0.04	0.448	0.13	**	0.24	0.31
	TIM-3 (HAVCR2)	0.236	***	0.205	***	0.28	***	0.046	0.85
Macrophage	CD68	0.084	0.091	0.02	0.702	0.19	***	0.22	0.37
	CD11b (ITGAM)	0.292	***	0.277	***	0.33	***	0	1
M1	INOS (NOS2)	0.06	0.23	0.056	0.286	0.11	*	0.21	0.39
	IRF5	0.153	**	0.151	**	0.14	**	0.11	0.65
	COX2 (PTGS2)	0.254	***	0.231	***	0.28	***	0.15	0.55
M2	CD16	0.292	***	0.27	***	0.33	***	0.033	0.89
	ARG1	0.009	0.857	0.029	0.579	-0.054	0.28	-0.17	0.48
	MRC1	0.296	***	0.283	***	0.34	***	0.16	0.51
	MS4A4A	0.261	***	0.23	***	0.29	***	0.074	0.76
TAM	CCL2	0.272	***	0.251	***	0.29	***	0.028	0.91
	CD80	0.188	***	0.151	**	0.25	***	0.13	0.6
	CD86	0.189	***	0.138	**	0.25	***	0.18	0.45
	CCR5	0.197	***	0.148	**	0.23	***	0.42	0.075
Monocyte	CD14	0.193	***	0.146	**	0.24	***	-0.021	0.93
	CD16 (FCGR3B)	0.187	***	0.174	***	0.33	***	-0.14	0.58
	CD115 (CSF1R)	0.215	***	0.168	**	0.27	***	0.17	0.48
Neutrophil	CD66b (CEACAM8)	0.115	*	0.132	*	0.091	0.068	-0.032	0.9
	CD15 (FUT4)	0.333	***	0.295	***	0.35	***	0.23	0.33
	CD11b (ITGAM)	0.292	***	0.277	***	0.33	***	0	1
Natural killer cell	XCL1	-0.034	0.489	-0.034	0.512	-0.035	0.48	0.47	*
	CD7	0.088	0.077	0.016	0.765	0.12	*	0.19	0.42
	KIR3DL1	0.047	0.347	0.018	0.724	0.074	0.14	0.4	0.09
Dendritic cell	CD1C (BDCA-1)	-0.017	0.734	-0.088	0.093	0.006	0.91	0.27	0.27
	CD141 (THBD)	-0.015	0.766	-0.046	0.379	0.086	0.085	-0.014	0.96
	CD11C (ITGAX)	0.233	***	0.209	0.055	0.27	***	-0.028	0.91

BLCA, bladder urothelial carcinoma; Tfh, follicular helper T cell; Th, T helper cell; Treg, regulatory T cell; TAM, tumour-associated macrophage; None, correlation without adjustment; Purity, correlation adjusted by purity; Tumour, correlation analysis in the tumour tissue of TCGA; Normal, correlation analysis in normal tissue of TCGA; Cor, R value of Spearman’s correlation. * *p*<0.05; ** *p*<0.01; *** *p*<0.001.

### KDELR2 expression was related with malignant biological features of BLCA cells

**Figure 8 -:**
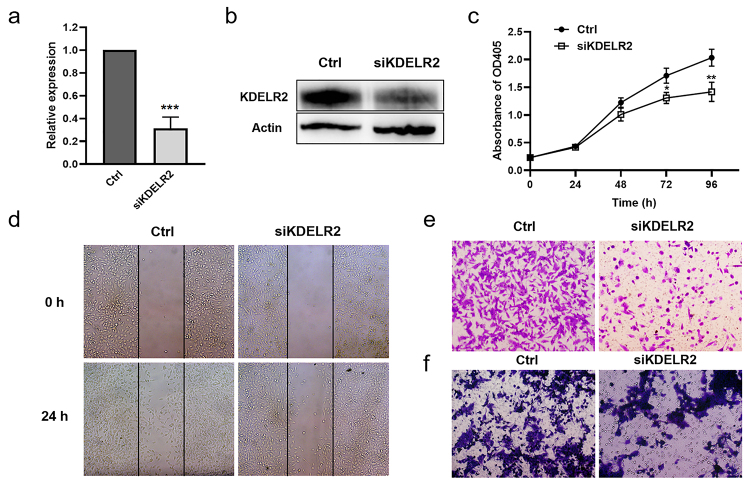
The effects of KDELR2 on the viability, migration and macrophage recruitment functions of BLCA cells. T24 cells were transfected with control siRNA or siRNA targeting KDELR2 for 48 h, and qRT‒PCR (a) and western blotting (b) were performed to detect the expression level of KDELR2. (c) Cell viability assays of siKDELR2 compared to the control over 4 days. *P<0.05, **P<0.01. Cell migration ability was evaluated by wound healing assays (d) and Transwell migration assays (e). The macrophage recruitment ability of the control cells versus the siKDELR2 cells in Transwell migration assays (f).

We then used T24 cells to analyse the effects of KDELR2 on the biological features of BLCA. We found that siRNA targeting KDELR2 significantly reduced KDELR2 expression (Figure 8a & b). The knockdown of KDELR2 by siRNA inhibited T24 cell proliferation (Figure 8c) and migration (Figure 8d & e). More importantly, knocking-down of KDELR2 impeded the recruitment of macrophages (Figure 8f), suggesting that KDELR2 might regulate BLCA pathogenesis by affecting immune cell infiltration.

## Discussion

BLCA is a common type of urinary tumour with characteristics of high incidence, high recurrence rate, and variable outcomes (Paz et al., 2014). There is a lack of reliable methods for predicting treatment response and guiding individualized treatment. Therefore, the identification of biomarkers to facilitate decision-making in diagnosis, prognosis and treatment is crucial. 

In addition to its involvement in chaperon retrieval (van Dijk et al., 2020), research work in recent years suggested that KDELRs may have other functions, such as signal transduction through the activation of Src family kinases, indicating that KDELRs may have regulatory capacity in tumorigenesis and progression. Indeed, the promotive effects of KDELR2 in several maliganant tumors have been reported (Liao et al., 2019; Bajaj et al., 2020; Mao et al., 2020). However, no research has been reported exploring the involvement of KDELR2 in BLCA.

In our study, a fully integrated bioinformatics analysis was performed to explore the possible biological function of KDELR2 in BLCA. KDELR2 expression was upregulated in various cancers, including BLCA. Furthermore, the overexpression of KDELR2 was related to poor clinicopathological features and a reduced OS. ROC curve analysis verified that KDELR2 is a biomarker with potential diagnostic value for BLCA. It is worth noting that increased KDELR2 expression is related with a poor prognosis, suggesting that KDELR2 may be an independent prognostic indicator for BLCA.

The tumour microenvironment (TME), which is a combination of extracellular matrix, mesenchymal cells, immune cells and inflammatory mediators, has a complex impact on the genesis and development of various malignant tumours (Chen et al., 2020). In this research, GO functional enrichment demonstrated that KDELR2-related DEGs were mainly enriched in biological functions associated with the organization of the extracellular matrix and other structures. Through GSEA, it was found that KDELR2 may be involved in PD-1 signalling, collagen degradation, cytokine‒cytokine receptor interactions, the syndecan1 pathway, degradation of the extracellular matrix, MET-activated PTK2 signalling and MET-promoted cell motility, indicating that KDELR2 may influence the biological process of extracellular matrix assembly and decomposition. Extracellular matrix degradation is an important process in the growth and invasion of malignant tumors (Paz et al., 2014), and previous research revealed that KDELR2 can potently enhance extracellular matrix degradation through activation of KDELR-Src pathway (Ruggiero et al., 2015) and enhancement of Golgi-mediated secretion of MMPs (Bajaj et al., 2020). 

The occurrence and progression of tumours also relies on the complicated functional association network between biological molecules. This research found that KDELR2 may influence genesis and development of BLCA through interactions with COPA, ARFGAP1, ARFGAP3, COPB1, and ARF1. Recently, an *in vitro* study showed that upregulation of COPA increased the vitality of breast cancer cells and promoted their invasion and migration (Peng et al., 2018). As for COPB1, it is positively related with PD-L1 in a number of malignant tumors including clear cell carcinoma of the kidney, sarcoma, gastric cancer, thyroid carcinoma and thymoma (Chen et al., 2021). As for ARF1, published research work indicated that it can inhibit the infiltration and activation of T cells in many cancers (Wang et al., 2020).

BLCA, a highly immunogenic malignant tumor, is often associated with a dysregulated immune response in the TME. The findings of this study indicate a positive correlation between KDELR2 expression and the infiltration levels of macrophages, Th2 cells, and Tregs. Macrophages and Th2 cells have tumorigenic properties, while Tregs can promote cancer progression through the modulation of immune surveillance and suppression of the antitumor immune response (Hurkmans et al., 2020). Furthermore, tumor-associated macrophages and Treg cells in the tumor tissue can directly inhibit the function of CD8+T cells, leading to reduced effectiveness of immunotherapy. Our findings suggest that KDELR2 may contribute to the development of BLCA by influencing immune infiltration in the TME. Inhibiting the expression of KDELR2 is likely to enhance the effectiveness of immunotherapy.

Although the involvement of KDELR2 in BLCA has been systematically analysed, our research still has some limitations. First, this study was performed based on analysis of public databases and *in vitro* experimental studies. Therefore, *in vivo* as well as experimental studies on clinical samples are required in future researches to verify the reliability of the present findings and elucidate the exact mechanism underlying the effects of KDELR2. Second, although our study revealed that the expression of KDELR2 is highly relevant to the prognosis of BLCA, further retrospective and prospective clinical trials are needed to validate these findings. Thirdly, the possibility that the DEGs identified in the present study are directly related with cancer progression rather than KDELR2 can not be denied. Further basic and clinical researches are required to elucidate the interaction between KDELR2 and other molecules and their functions in development of BLCA. 

## Conclusions

The present study revealed an enhanced expression of KDELR2 in BLCA and such an increased expression was remarkably related with unfavourable clinicopathological features and prognosis. ROC analysis indicated that KDELR2 could have diagnostic value in discriminating BLCA tissue from normal tissue. KDELR2 could also be a useful biomarker to predict the outcomes of BLCA patients. KDELR2 may contribute to the progression of BLCA by modulating immune infiltration in TEM.

## Supplementary material

The following online material is available for this article:

Figure S1 - KDELR2-related protein network in BLCA. Annotation of KDELR2-interacting proteins and their co-expression scores are shown.
