# Supplementary material for: KDELR2 as a diagnostic and prognostic biomarker of bladder urothelial carcinoma and its correlation with immune infiltration
Source: Genet Mol Biol. 2023 Sep 25;46(3):e20230002. doi: 10.1590/1678-4685-GMB-2023-0002 (PMC10548500; doi:10.1590/1678-4685-GMB-2023-0002)
Supplement: Figure S1 - [file 1415-4757-GMB-46-3-e20230002-s1.pdf]

Genetics and Molecular Biology
https://doi.org/10.1590/1678-4685-GMB-2023-0002

Supplementary Material to "KDELR2 as a diagnostic and prognostic biomarker of bladder urothelial carcinoma and its correlation with immune infiltration"

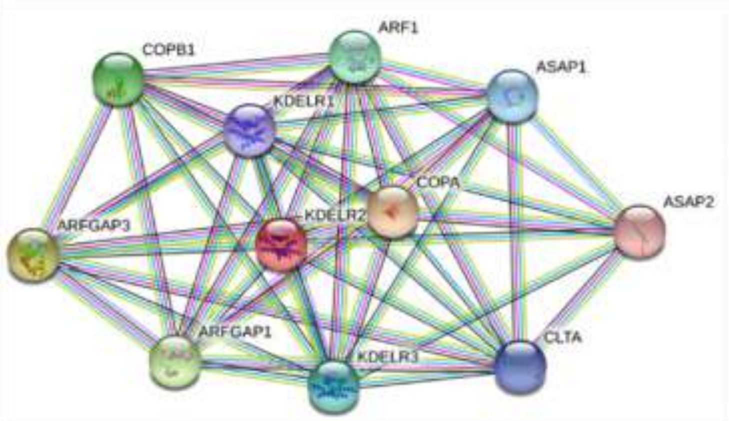

Gene symbol	Annotation	Score
COPA	Coatomer subunit alpha	0.937
ARFGAP1	ADP -ribosylation factor GTPase-activating protein 1	0.934
ARFGAP3	ADP -ribosylation factor GTPase -activating protein 3	0.934
COPB1	Coatomer subunit beta	0.929
ARF1	ADP-ribosylation factor 1	0.925
KDELR3	ADP-ribosylation factor GTPase-activating protein 3	0.919
ASAP1	Arf-GAP with SH3 domain, ANK repeat and PH domain-containing protein 1	0.915
CLTA	Clathrin light chain A	0.906
KDELR1	ADP-ribosylation factor GTPase-activating protein 1	0.906
ASAP2	Arf-GAP with SH3 domain, ANK repeat and PH domain-containing protein 2	0.904

Figure S1 - KDELR2-related protein network in BLCA. Annotation of KDELR2-interacting proteins and their co-expression scores are shown.
